# Expression level of CD117 (KIT) on ovarian cancer extracellular vesicles correlates with tumor aggressiveness

**DOI:** 10.3389/fcell.2023.1057484

**Published:** 2023-02-16

**Authors:** Polina V. Shnaider, Irina Yu. Petrushanko, Olga I. Aleshikova, Nataliya A. Babaeva, Lev A. Ashrafyan, Ekaterina I. Borovkova, Julia E. Dobrokhotova, Ivan M. Borovkov, Victoria O. Shender, Elena Khomyakova

**Affiliations:** ^1^ Center for Precision Genome Editing and Genetic Technologies for Biomedicine, Federal Research and Clinical Center of Physical-Chemical Medicine of Federal Medical Biological Agency, Moscow, Russia; ^2^ Laboratory of Molecular Oncology, Federal Research and Clinical Center of Physical-Chemical Medicine of the Federal Medical and Biological Agency, Moscow, Russia; ^3^ Faculty of Biology, Lomonosov Moscow State University, Moscow, Russia; ^4^ Engelhardt Institute of Molecular Biology, Russian Academy of Sciences, Moscow, Russia; ^5^ National Medical Scientific Centre of Obstetrics, Gynaecology and Perinatal Medicine named after V.I. Kulakov, Moscow, Russia; ^6^ Department of Obstetrics and Gynecology, Faculty of Medicine, Pirogov Russian National Research Medical University, Moscow, Russia; ^7^ Department of Oncology and Hematology, RUDN University, Moscow, Russia; ^8^ Laboratory of Molecular Oncology, Shemyakin-Ovchinnikov Institute of Bioorganic Chemistry of the Russian Academy of Sciences, Moscow, Russia; ^9^ Exosome Analytics, Evry, France

**Keywords:** extracellular vesicles, EVS, ovarian cancer, chemoresistance, prognositic biomarkers, liquid biopsy, CD117, EpCAM

## Abstract

Ovarian cancer is known to be the most lethal malignancy among all gynecological cancers affecting a large number of women worldwide. The treatment of ovarian cancer is challenging due to the high recurrence rate of the disease and is further complicated by acquired chemoresistance. Most ovarian cancer deaths are the result of the metastatic spread of drug-resistant cells. The theory of cancer stem cells (CSC) suggests that both tumor initiation and progression are driven by a population of undifferentiated capable of self-renewal, tumor initiation and development of chemoresistance. The CD117 mast/stem cell growth factor receptor (KIT) is the most commonly used marker for ovarian CSCs. Here, we analyze the correlation between CD117 expression and histological tumor type in ovarian cancer cell lines (SK-OV-3 and MES-OV) and in small/medium extracellular vesicles (EVs) isolated from the urine of ovarian cancer patients. We have demonstrated that the abundance of CD117 on cells and EVs is correlated with tumor grade and therapy resistance status. Moreover, using small EVs isolated from ovarian cancer ascites, it was shown that recurrent disease is characterized by a much higher abundance of CD117 on EVs than primary tumor.

## Introduction

Ovarian cancer (OC) is the most lethal gynecologic malignancy. Even after surgical cytoreduction in combination with platinum-based chemotherapy, the 5-year survival rate for ovarian cancer patients remains 30%–40% and about 80% of patients will have a recurrence (van Zyl et al., 2018; Berek et al., 2021). The main reason for the low survival rate of patients with ovarian cancer is the late cancer diagnosis as well as acquisition of therapy resistance by relapsed tumors (Lheureux et al., 2019). It has been recently shown that chemotherapy can provoke the spread of therapy-resistant clones or activation of compensatory signaling pathways, which allows the tumor cells to cope with damage and initiate tumor repopulation (Izar et al., 2020; Shnaider et al., 2020; Kan et al., 2022; Zhang et al., 2022). Thus, there is a need to develop new methods for prediction of tumor aggressiveness and reliable early detection of cancer progression along with new chemotherapy regimens. The Cancer Stem Cell hypothesis posits that tumor initiation and progression are driven by a rare subpopulation of non-differentiated cells. These cells carry mesenchymal markers, which can be used as biomarkers of tumor aggressiveness, prediction of treatment success and prognosis (Blassl et al., 2016; Harris et al., 2021).

It has been repeatedly shown that CD117 (a product of the c-KIT gene) is expressed in many aggressive cancers (including ovarian cancers) as well as in recurrent and resistant tumors and predicts poor survival of patients (Chau et al., 2013; Conic et al., 2015; Stemberger-Papić et al., 2015; Yang et al., 2017; Foster et al., 2018; Fang et al., 2020). Along with other tyrosine kinases, CD117 participates in important processes of cancer progression: proliferation, metabolism, cell growth, regulation of cell migration, differentiation and apoptosis (Foster et al., 2018).

Another common cancer marker is Epithelial Cell Adhesion Molecule (EpCAM). However, the role of EpCam in tumor initiation, progression, and therapy resistance in not well established. Despite the role of EpCam as mediator of epithelial cell-cell adhesion, some works mention it as a marker of tumor initiating cells (Gires et al., 2009). However, during chemotherapy, epithelial tumor cells often undergo an epithelial-mesenchymal transition. Such cells acquire a more mesenchymal phenotype, which leads to the loss of the EpCAM epithelial marker (Hyun et al., 2016). Thus, EpCam does not reflect the phenotype of cancer-initiating cells and further studies of the role of this glycoprotein in tumor initiation and progression, as well as the possibility of its application in diagnostics, is required.

The standard approach for tumor profiling is tissue biopsy where molecular markers are analyzed in the tumor punction (Mari et al., 2019). However, in the case of ovarian cancer, tissue biopsy has severe limitations since it requires serious surgical interventions that is not well tolerated by patients and cannot be performed several times during the course of the therapy. The liquid biopsy is the most suitable method for biomaterial sampling (Mari et al., 2019). Initially, only circulating tumor cells and circulating tumor DNA were studied in the context of liquid biopsies. Extracellular vesicles (EVs) have been attracting significant research interest because they constitute a promising tool for important medical applications including liquid biopsy tests (Poulet et al., 2019; Zhou et al., 2020; Yu et al., 2022). EVs are found in body fluids and in the cultured media of different cell lines. They are enriched with proteins, lipids and nucleic acids (Zhang and Yu, 2019; Yokoi and Ochiya, 2021) which reflect the composition of the cell of origin. EVs are involved in various cellular processes including intercellular communication and immune regulation (Zhang and Yu, 2019; Yokoi and Ochiya, 2021). EV-based liquid biopsies would allow non-invasive profiling of protein, RNA and DNA tumor markers, monitoring the modifications in tumor phenotype during therapy, prediction of tumor response to therapy and post-treatment follow-up of the patients.

One of the features of advanced stages of ovarian cancer is the formation of ascitic fluid in the abdominal cavity. Ascites contain proteins, lipids, nucleic acids and metabolites as well as extracellular vesicles secreted by both tumor cells and associated stromal and immune cells (Ahmed and Stenvers, 2013; Shender et al., 2014; Rickard et al., 2021). Another body fluid of great value for liquid biopsies is urine since it contains large quantities of extracellular vesicles that allow non-invasive profiling of urogenital tumors. Moreover, urine-based diagnostics allows the profiling of early-stage tumors prior to the formation of ascitic fluid.

In the present work, we performed an analysis of the expression of mesenchymal marker CD117 and cell adhesion glycoprotein EpCam in tumor cells and the abundance of these markers on the surface of tumor-derived extracellular vesicles. We analyzed two ovarian cancer cell lines MES-OV and SK-OV-3 and clinical samples from patients with ovarian cancer with different histological types and grades to study the correlation between CD117/EpCam abundance and aggressiveness of the tumors.

## Materials and methods

### Cell culture

Human ovarian cancer cell line SK-OV-3 (ATCC^®^ HTB-77™) and human cystadenocarcinoma cell line MES-OV (ATCC^®^ CRL-3272™) were maintained in Dulbecco’s Modified Eagle Medium (DMEM; Gibco) and Dulbecco’s Modified Eagle Medium/Nutrient Mixture F-12 (DMEM/F-12; Gibco), accordingly, supplemented with 10% fetal bovine serum (FBS; Gibco), 2 mM glutamine (GlutaMAX; Gibco), and penicillin/streptomycin (1%) (Gibco), at 37°C in a humidified atmosphere containing 5% CO2.

### Flow cytometry analysis of cells

SK-OV-3 and MES-OV cells were collected and fixed with 4% PFA, blocked with 2% FBS solution and incubated overnight with primary antibodies: CD117-PE (BD Pharmigen), EpCam (Abcam), CD9-FITC (BD Pharmigen). Next, cells were washed three times with PBS and incubated with secondary antibodies if needed for 1 h (Goat anti-Rabbit IgG, Alexa 647 (Invitrogen) for EpCam). Stained samples were analyzed with NovoCyte Flow Cytometer (ACEA Biosciences). The data were analysed with Kaluza software (Beckman Coulter, United States).

### Isolation of extracellular vesicles

At 90% confluence SK-OV-3 and MES-OV cells were washed 3 times with PBS and maintained in 30 mL FBS-free, phenol-red-free DMEM (Gibco). After 24 h cell culture supernatants (28 mL per 175 cm^2^ flasks) were harvested and centrifuged at 500 g for 5 min at 14°C (Eppendorf Centrifuge 5804 R, A-4-44 swinging-bucket rotor) to pellet dead cells followed by centrifugation at 4,000 g for 20 min at 4°C in the same rotor to eliminate debris and large vesicles. Small and medium extracellular vesicles were collected by ultrafiltration of supernatants with 100-kDa Amicon Ultra-15 Centrifugal Filter Units (Millipore) according to manufacturer protocol.

### NTA analysis

Measurements of particle size distribution (PSD) and concentration were made with Nanosight NS300 instrument (Malvern) based on Nanoparticle Tracking Analysis (NTA). Measurements were performed with 405 nm, 65 mW laser and high sensitivity sCMOS camera. Samples were diluted with particle-free PBS (pH = 7.4) to reach the optimal concentration for NTA according to ASTM E2834–12. All measurements were made under the same camera settings (Shutter: 1,206-1,300, Gain: 366-512, camera level: 16, time: 60 s) and processing conditions (NTA 3.3 build 3.3.203, Detection Threshold: 5). Measurements were done in several repeats (3–5) to collect at least 4,000 particles in total. To obtain the joint histogram of PSD for multiple measurements, single particle diameters and track lengths from each measurement were collected into a global table and binned with weights proportional to track lengths. A decrease of the total vesicle concentration during the storage (at 4°C) was taken into account.

### Patient samples

Ascitic fluids from ovarian cancer patient were obtained from the Russian Scientific Center of Roentgen Radiology (Moscow, Russia), Ministry of Healthcare of the Russian Federation. The study was approved by the Ethics Committee of the Russian Scientific Center of Roentgen Radiology (agreement and protocol no. 30-2018/E from 13 November 2018), and written informed consent was obtained from all the patients who participated. The ascites of primary tumor (ovarian papillary cystadenocarcinoma) was collected before chemotherapy. The relapsed ascites were collected after six courses of neoadjuvant paclitaxel/carboplatin chemotherapy. Fresh ascitic fluids were centrifuged at 300 g for 15 min to pellet cells and cell debris (Eppendorf Centrifuge 5804 R, A-4-44 swinging-bucket rotor). The supernatants were collected and stored at −80°C until further processing.

For EVs isolation from ovarian cancer ascites samples, 500 µL of the ascitic fluids were centrifuged at 500 g for 15 min, then at 10,000 g for 30 min in an F-45–24-11 rotor (Eppendorf) at 4°C. Then, the upper-phase containing largest lipoproteins was removed, 12 mL of PBS buffer was added to supernatant followed by concentration with 100-kDa Amicon Ultra-15 Centrifugal Filter Units (Millipore) and washing with PBS buffer using the same filter unit.

Urine samples from ovarian cancer patients and individuals without cancer were obtained from Department of Obstetrics and Gynecology, Faculty of Medicine, Pirogov Russian National Research Medical University (Figure 4A). Written informed consent was obtained from all the patients who participated. The urine of cancer patients was collected before treatment. To isolate extracellular vesicles, urine was centrifuged at 4,000 g for 20 min at 4°C in the A-4-44 swinging-bucket rotor, concentrated with 100-kDa Amicon Ultra-15 Centrifugal Filter Units (Millipore) and washed twice with PBS buffer.

### Flow cytometry analysis of extracellular vesicles

Profiling of CD81, CD117 and EpCam markers on EVs was performed with ExoSence FACS kit beta (Exosome Analytics, France). Pre-purified EVs (the EVs quantity per reaction is shown in Table 1) were incubated with antiCD9 coated magnetic beads for 18 h followed by incubation of EVs-bead complexes with CD81-PE, CD117-PE or EpCam-PE antibodies according to the manufacturer’s protocol and analysed either with BD LSRFortessa™ Cell Analyzer or CytoFLEX cytometer (Beckman Coulter). The data were analysed with Kaluza software (Beckman Coulter, United States).

**TABLE 1:** The quantity of EVs (for cell lines) and volume of body fluids (for clinical samples) taken for each reaction.

	Protein markers		
Source of EVs	CD81	CD117	EpCam
MES-OV	4 × 10^7^ EVs	10^9^ EVs	10^9^ EVs
SK-OV-3	1.3 × 10^8^ EVs	3.3 × 10^9^ EVs	3.3 × 10^9^ EVs
Ascitic fluids	100 µL	150 µL	150 µL
Urine	2 mL	13 mL	13 mL

## Results

### Expression of CD117 and EpCam proteins in SK-OV-3 and MES-OV cells

**FIGURE 1:**
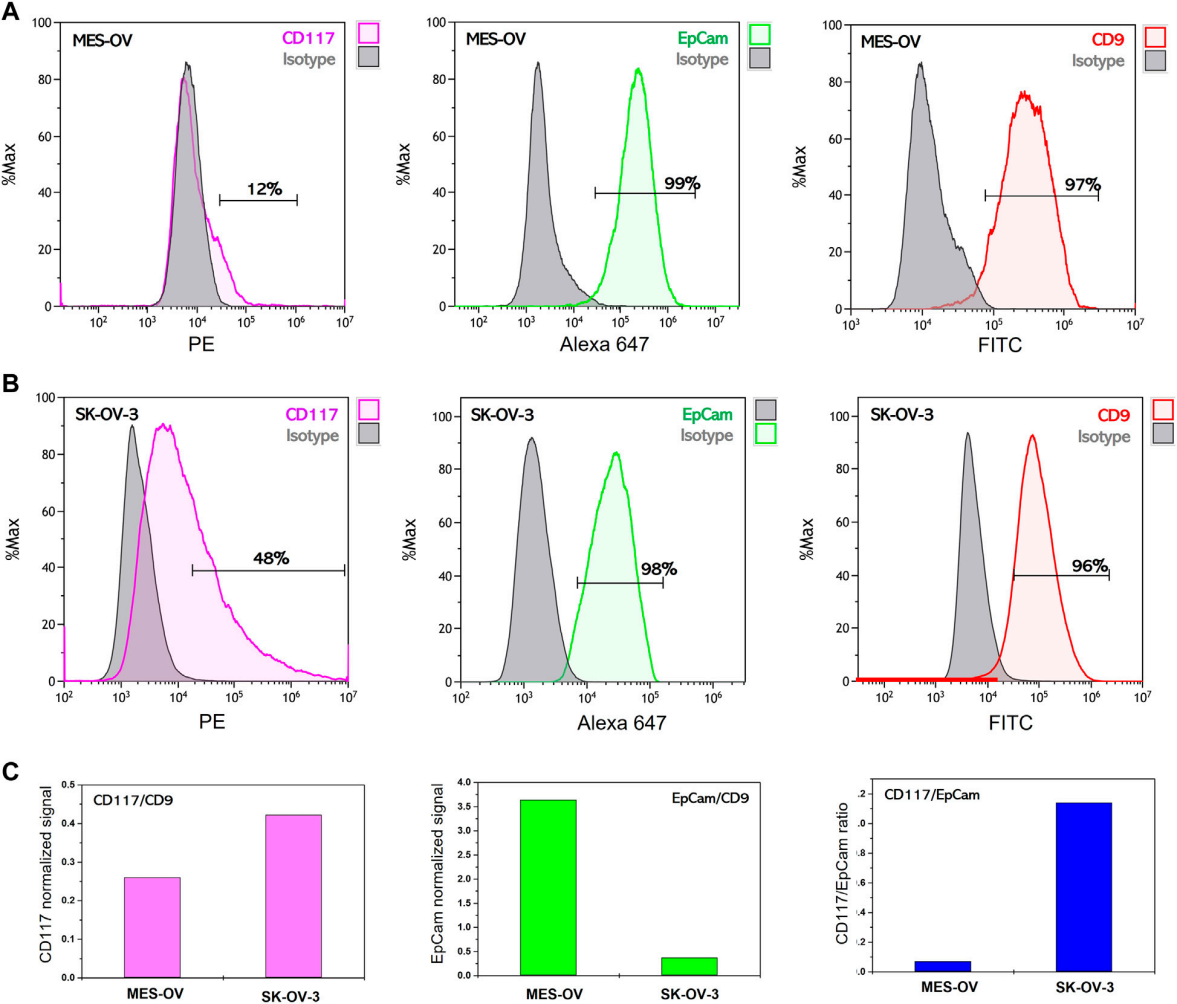
Flow cytometry analysis of membrane surface protein expression on MES-OV and SK-OV-3 cells. Flow histograms of CD117-PE, EpCam-Alexa 647 and CD9-PE staining of MES-OV **(A)** and SK-OV-3 cells **(B)**. **(C)** Ratios of median fluorescent signals.

Elevated levels of receptor tyrosine kinase CD117 expression have been shown to be associated with chemoresistance of ovarian cancer cells to platinum-based drugs (Tomao et al., 2013). We assessed the expression level of CD117 in two ovarian cancer cell lines: SK-OV-3 and MES-OV. Both cell lines were established from ascitic fluids of ovarian cancer patients (ATCC HTB-77™ and ATCC CRL-3272™). SK-OV-3 cells are a well-known ovarian cancer model with an aggressive phenotype. Despite the absence of available information concerning the tumor grade, SK-OV-3 cells demonstrate resistance to platinum-based drugs, have a high invasive potential and tumorigenicity among the other ovarian cancer cell lines (Hallas-Potts et al., 2019). MES-OV cells have been little studied. They represent moderately differentiated (grade 2) ovarian tumor but have traits of a mesenchymal phenotype. Figure 1A represents flow histograms for CD117, EpCam and CD9 staining of MES-OV and SK-OV-3 cells. As follows from the results, EpCam expression is observed in more than 90% of the cells. However, it is important to note that EpCam median specific fluorescent signal is 10 times lower for SK-OV-3 cells as compared to MES-OV cells (2 × 10^5^ a. u. for MES-OV cells vs. 2 × 10^4^ a. u. for SK-OV-3 cells; Figures 1A, B). An explication of this discrepancy could be the fact that the quantity of EpCam antigens is essentially lower in SK-OV-3 cells than in MES-OV cells. The opposite is observed for CD117 expression. As shown in Figure 1, about half (47%) of SK-OV-3 cells are CD117-positive, compared with only 12% of MES-OV cells. Thus, taking into consideration EpCam and CD117 expression pattern, one can conclude that SK-OV-3 cells have a more pronounced mesenchymal-like phenotype than MES-OV cells, which corresponds well to highly aggressive properties of SK-OV-3 cells.

Expression of the CD9 antigen, which is a common EV marker, was also analyzed in MES-OV and SK-OV-3 cells. At least 97% of MES-OV cells and 96% of SK-OV-3 cells are CD9-positive, and the median value of antiCD9-PE fluorescence is rather close for both cell lines (Figure 1A). To move towards the application of EVs in liquid biopsy tests for the profiling of tumor markers, it is necessary to prove the correspondence between the abundance of marker proteins in EVs and the expression pattern of these markers in the donor cells.

### Profiling of EVs isolated from SK-OV-3 and MES-OV cell cultures

#### Characterization of MES-OV and SK-OV-3- derived EVs

EVs were isolated from cultured media of MES-OV and SK-OV-3 cells by differential centrifugation followed by ultrafiltration (as described in Material and Methods). Centrifugation conditions were chosen to obtain a mixed population of small and medium-sized EVs [cut-off size 200 nm according to (Livshits et al., 2015)]. Size distribution and concentration of the MES-OV and SK-OV-3 EVs were analyzed by nanoparticle tracking analysis (NTA) The EVs size distribution corresponds well to the theoretical prediction for both cell lines (Supplementary Figure S1). The measured EVs’ mean size is 101±3.5 nm and 126.6±4.4 nm for MES-OV and SK-OV-3 vesicles, respectively.

#### Profiling of CD117 and EpCam on SK-OV-3 and MES-OV derived extracellular vesicles

**FIGURE 2:**
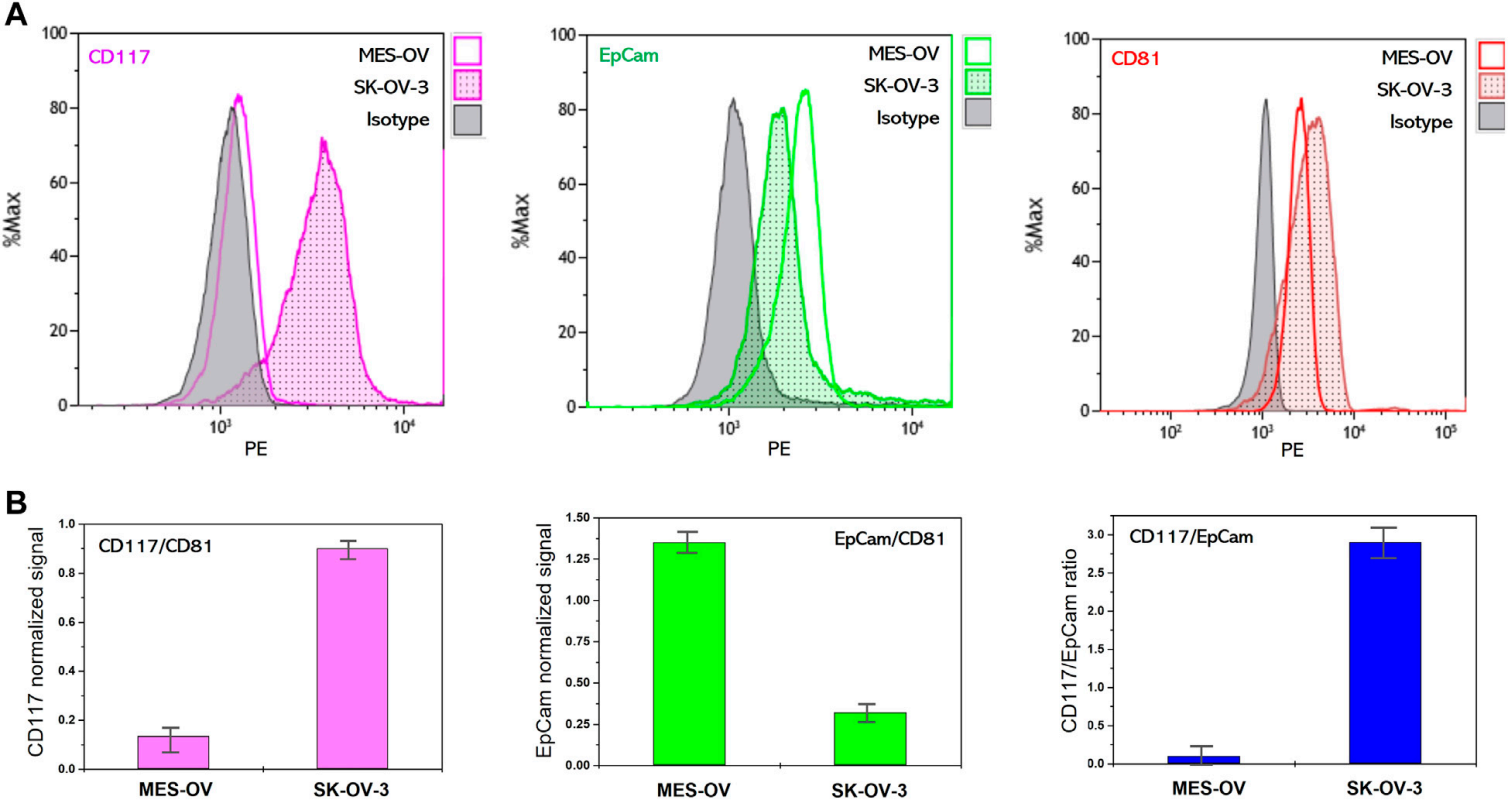
Flow cytometry analysis of membrane protein abundance on MES-OV and SK-OV-3-derived EVs. **(A)** Flow histograms of CD117-PE, EpCam-PE and CD81-PE staining of MES-OV and SK-OV-3-derived EVs immunoprecipitated on antiCD9 magnetic beads. **(B)** Ratios of median fluorescent signals. Error bars correspond to the standard deviation of median PE fluorescence calculated from the two measurements.

To prove the correspondence of the CD117 and EpCam abundance on EVs to the profiles of CD117 and EpCam expression in the cells of origin, we analyzed the expression of these markers on SK-OV-3- and MES-OV-derived EVs. To approach real liquid biopsy test conditions where vesicle concentration varies between the clinical samples and is *a priori* unknown, in our experiments the concentrations of SK-OV-3- and MES-OV-derived EVs were also different (Materials and Methods section, Table 1). The abundance of CD81, CD117, and EpCam antigens were analyzed by flow analysis of EVs immunoprecipitated to antiCD9 coated magnetic beads and stained with the antibody of interest. AntiCD9 beads were chosen for EV capture since the CD9 antigen is widely represented in many ovarian cancer tumors including MES-OV and SK-OV-3 cell lines (Hwang et al., 2012; Lorico et al., 2021; Figure 1). Figure 2A shows flow histograms of CD81-PE, CD117-PE and EpCam-PE stained MES-OV and SK-OV-3-derived extracellular vesicles.

The difference in CD81 fluorescence values reflects the difference in input of MES-OV and SK-OV-3 vesicles. A CD117 signal is reliably detected on MES-OV extracellular vesicles, however the value of the signal is rather low. Contrary, a strong CD117 signal is observed for SK-OV-3-derived EVs. The opposite pattern was observed for EpCam expression. EpCam signal corresponding to MES-OV-derived vesicles is significantly higher than for SK-OV-3 EVs (Figure 2A).

However, since MES-OV and SK-OV-3 EVs input was different, direct comparison of the CD117 and EpCam signals between MES-OV- and SK-OV-3-derived vesicles is not possible and signals normalization is required. One of the possible approaches is normalization to EVs concentration measured by NTA. However, even though such an approach is common is laboratory practice, it is much less applicable in real liquid biopsy tests as NTA measurements strongly complicate the diagnostic procedure. In our study, we normalize CD117 and EpCam signal value obtained for MES-OV and SK-OV-3 vesicles to the value of the CD81 signal. Since tetraspanins CD9 and CD81 are believed to be common EVs markers, such a ratio reflects the portion of CD117-positive vesicles in total EVs population. As shown in Figure 2B, CD117 and EpCam normalized values inversely correlate in MES-OV- and SK-OV-3-derived EVs that fully corresponds to the profiles of original cells. This observation confirms correspondence of CD117 and EpCam expression profiles on cells and extracellular vesicles that allows the application of EVs in liquid biopsy tests for profiling of CD117 and EpCam markers on tumors.

### CD117 and EpCam profiling of extracellular vesicles isolated from clinical samples

#### Profiling of CD117 and EpCam on extracellular vesicles isolated from ascites of primary ovarian cancer and relapsed tumor

We analyzed the evolution of the abundance of CD117 and EpCam markers on EVs in the course of the development of post-treatment relapsed ovarian tumor. In our pilot experiment, we performed comparative analysis of CD117, EpCam and CD81 abundance on EVs isolated from ascitic fluids of the patient with primary tumor (ovarian papillary cystadenocarcinoma) and of the same patient with post-treatment tumor relapse.

**FIGURE 3:**
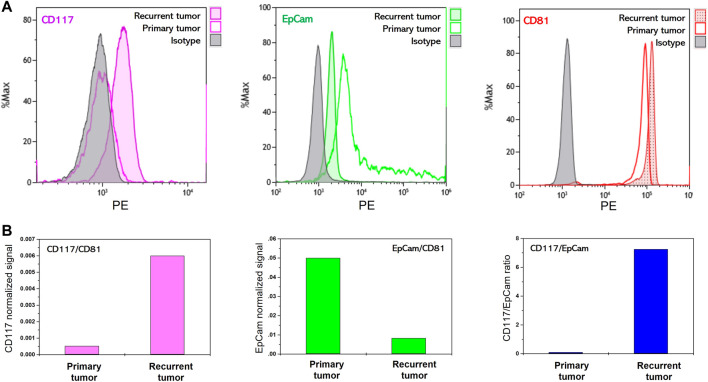
Flow cytometry analysis of membrane protein abundance on EVs isolated from ascites of ovarian cancer patient. **(A)** Flow histograms of CD117-PE, EpCam-PE and CD81-PE staining EVs immunoprecipitated on antiCD9 magnetic beads. **(B)** Ratios of median fluorescent signals.

Figure 3A shows flow histograms corresponding to antiCD81-PE, antiCD117-PE and antiEpCam-PE staining of EVs immunocaptured with antiCD9 magnetic beads. As follows from the data, very high CD81 signal was observed for EVs isolated from just 100 µL of ascites of both primary and relapsed tumors. Slightly higher level of CD81 signal corresponding to EVs isolated from recurrent ascites reveals their higher concentration in the ascitic fluid of relapsed tumor. CD117 signal was undetectable on primary tumor EVs, however the CD117 signal is reliably detectable on relapsed tumor EVs. The opposite is observed for the expression of EpCam. EVs from primary tumor ascites showed a higher EpCam signal than EVs isolated from relapsed tumor ascites. Thus, as shown in the profiling data, the pattern of CD117 and EpCam abundance on primary tumor EVs corresponds to the profile of MES-OV cells and extracellular vesicles. On the other hand, recurrent tumor acquires an expression signature characterized by high CD117 and low EpCam expression as was observed for highly aggressive SK-OV-3 cells. These observations are in good agreement with the statement that relapsed ovarian tumors acquire mesenchymal properties and become more aggressive than primary tumors (Pastushenko et al., 2018).

#### Profiling of CD117 on extracellular vesicles isolated from urine of ovarian cancer patients and healthy donors

**FIGURE 4:**
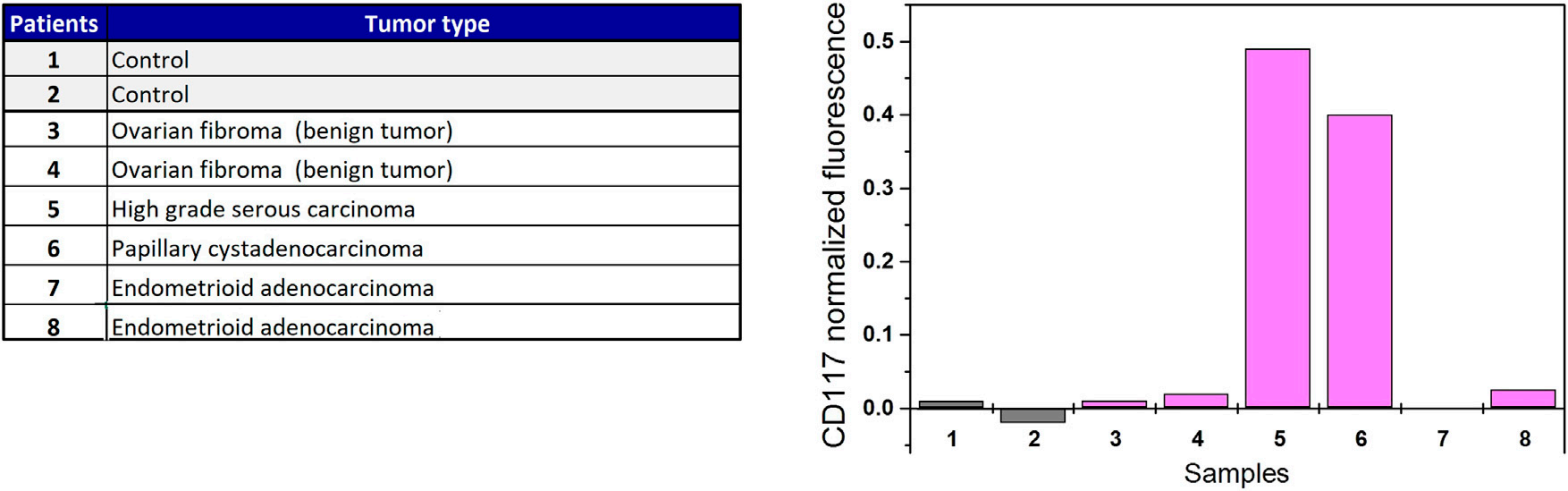
CD117 profiling of EVs isolated from urine of ovarian cancer patients and individuals without cancer.

CD117 abundance was analyzed on EVs isolated from the urine of patients with histologically characterized ovarian tumors and healthy donors (4 malignant and 2 benign tumors, Figure 4A). To estimate the proportion of CD117-positive EVs, normalization to the value of CD81 signal was applied (Figure 4B). As follows from the data, remarkable CD117 expression is detected only in two clinical samples. These samples correspond to patients with high grade serous ovarian carcinoma and papillary serous cystadenocarcinoma. No CD117 expression signal is observed either for healthy donors, or for patients with benign fibroma tumors and endometrioid adenocarconoma.

## Discussion

The present work aimed at analyzing the abundance of mesenchymal marker CD117 and epithelial cell adhesion molecule (EpCam) on the surface of extracellular vesicles derived from ovarian tumors. Using ovarian cancer cell line models, we demonstrated the increased expression of CD117 in highly aggressive SK-OV-3 cells. On the contrary, the MES-OV cells, which corresponds to a middle-grade ovarian tumor, contains only a minor population of CD117-positive cells. These results are in good agreement with the statement that CD117 expression is associated with tumor aggressiveness. Interestingly, although all cell populations in MES-OV and SK-OV-3 cell lines stained positive for EpCam, the median value of the EpCam signal was significantly lower in SK-OV-3 cells, which can be explained by a lower quantity of EpCam antigens being present on the membrane of SK-OV-3 cells. The same effect was observed in the MDA-MB-231 cell line originated from an aggressive triple negative breast tumor (Bragina et al., 2022). Thus, at least in some cancers, the value of EpCam expression does not correlate with aggressiveness of the tumor and its role in cancer biology needs to be further studied.

To answer the question whether EVs could be used in liquid biopsy tests for non-invasive tumor profiling, it is necessary to prove that the expression profile of cancer markers on tumor cells matches the abundance of these markers on tumor-derived EVs. Using highly sensitive FACS kit, we succeeded in detecting both CD117 and EpCam antigens on the surface of SK-OV-3- and MES-OV-derived extracellular vesicles. Moreover, we demonstrated that the profiles of CD117 and EpCam expression in donor cells and their EVs are identical when the fluorescence signals were normalized to vesicle concentration.

We applied a similar approach for the profiling of EVs isolated from physiological fluids. We analyzed the abundance of CD117 on EVs isolated from the urine of patients with different histological types of ovarian tumors and healthy women. No CD117 signal was detected in control samples. Among the patients with benign and malignant tumors, the EVs from serous ovarian carcinoma and papillary serous cystadenocarcinoma gave a strongly positive CD117 signal. The abundance of CD117 on EVs isolated from urine of patients with highly invasive serous ovarian carcinoma is not surprising since these tumors are known to be highly invasive with a documented mesenchymal signature, particularly with high CD117 expression (Foster et al., 2018). On the other hand, the papillary serous cystadenocarcinoma is a less common and much less studied disease than the serous ovarian carcinoma. Particularly, to our knowledge, the CD117 expression is not characteristic of either papillary serous cystadenocarcinoma tumor samples or established papillary serous cystadenocarcinoma cell lines. Generally, papillary serous cystadenocarcinoma is considered a low-grade tumor, however, some works demonstrate its increased invasive potential and resistance to treatment [early post-treatment tumor relapse is described in (Kaku et al., 2004)]. Thus, the screening of papillary serous cystadenocarcinoma for CD117 expression may contribute to a better understanding of the behavior of this type of tumor.

We also analyzed the EVs isolated from ascitic fluids of the same patient with a primary tumor (ovarian papillary cystadenocarcinoma, before treatment) and then after early recurrence (after six courses of chemotherapy). On the EVs from the primary tumor, no CD117 signal was detected. However, the EVs from the recurrent tumor carry essential quantity of CD117 antigen to be reliably detected. The described clinical case supports the hypothesis that a very small population of tumor-initiating cells, including CD117-positive cells, can survive during the treatment and give rise to an aggressive recurrent tumor therapy resistant phenotype (which led to the death of the patient). Thus, a novel treatment approach targeting cancer-initiating cells and particularly CD117-positive tumor cells could improve outcomes. Moreover, the screening of EVs for CD117 abundance could be potentially used for diagnostics of tumor relapse during post-treatment follow-up of the patients.

In this study we performed normalization of CD117 and EpCam signals to the value of the CD81 signal. However, the most effective approach in diagnostics is when the “positive” marker is normalized to the “negative” marker (Erdbrügger et al., 2021). That means if a diagnostic test is aimed at distinguishing between mesenchymal and epithelial tumors, the ratio between mesenchymal and epithelial markers must be considered. If we consider EpCam as an exclusively epithelial marker and calculate the signal ratio CD117/EpCam, the difference between SK-OV-3 and MES-OV cell lines, as well as between primary and relapsed ovarian tumors, is more pronounced. However, extended studies of the role of EpCam in cancer progression is required before its expression could be attributed to aggressive or non-aggressive tumors.

## Data Availability

The original contributions presented in the study are included in the article/Supplementary Material, further inquiries can be directed to the corresponding author.
