# Supplementary material for: Expression level of CD117 (KIT) on ovarian cancer extracellular vesicles correlates with tumor aggressiveness
Source: Front Cell Dev Biol. 2023 Feb 16;11:1057484. doi: 10.3389/fcell.2023.1057484 (PMC9978408; doi:10.3389/fcell.2023.1057484)
Supplement: Supplementary file 1 [file Image1.pdf]

Supplementary materials

Expression level of CD117 (KIT) on ovarian cancer extracellular vesicles correlates with tumor aggressiveness

Polina V. Shnaider, Irina Yu. Petrushanko, Olga I. Aleshikova, Nataliya A. Babaeva, Lev A. Ashrafyan, Ekaterina I. Borovkova, Julia E. Dobrokhotova, Ivan M. Borovkov, Victoria O. Shender and Elena Khomyakova

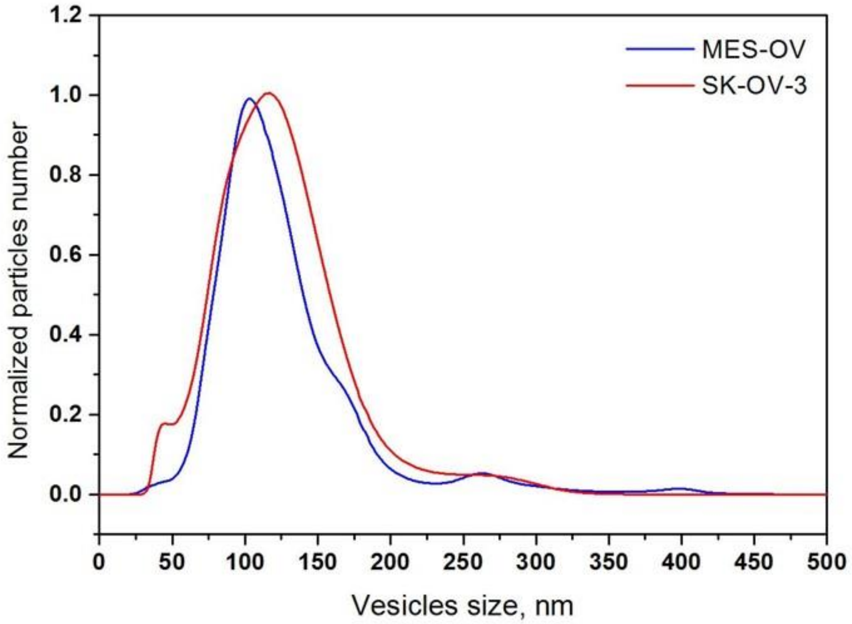

FIGURE 1S. NTA profiles of EVs isolated from MES-OV and SK-OV-3 cell cultures.
